# State of Lunacy in England

**Published:** 1851-01-01

**Authors:** 


### Art. VI.—STATE OF LUNACY IN ENGLAND.

We have before us the fifth annual report of the commissioners in lunacy, ordered to be printed by the House of Commons, on the 15th of August, 1850. By it we perceive that the number of insane persons confined in asylums, hospitals, and licensed houses, on the 1st of January, 1850, was 7140, of this number there were in the county and borough asylum—

| | Males | Females | Total |
|---|---|---|---|
| Private patients . | 120 | 112 | 232 |
| Pauper patients . | 3150 | 3758 | 6908 |

Found lunatic by inquisition, 5; 4 males and 1 female.—Criminal lunatics, 359; viz. 117 males and 31 females. Patients chargeable to counties and boroughs, 804; viz. 359 male and 445 females. The number of insane persons confined in hospitals, amounted to 1208; of this number, 865 were private patients; viz. 412 males, and 453 females; paupers 343; males 175, females 168. Found lunatic by inquisition, 17; females 3, males 14; criminal patients in hospitals, 5 males. Chargeable to counties and boroughs, 26; 14 males and 12 females.

The number of patients in the metropolitan licensed houses is 2945.

| Private | | Total | Paupers | |
|---|---|---|---|---|
| Males . . | 575 | 1120 | 737 | 1825 |
| Females . . | 546 | | 1,088 | |

Found Lunatic by Inquisition.

| | | |
|---|---|---|
| Males . . . | 59 | 96 |
| Females . . . | 37 | |

Criminal patients, 32; males 25, females 7. Private patients chargeable to counties and boroughs, 88; viz. 32 males, and 50 females.

*Provincial Licensed Houses.*

| Number of patients (private) | | | Paupers | |
|---|---|---|---|---|
| Males . | 800 | 1557 | 1106 | 2229 |
| Females . | 757 | | 1123 | |

Found Lunatic by Inquisition.

| | | |
|---|---|---|
| Males . . . . . | 76 | 120 |
| Females . . . . . | 44 | |

### SUMMARY.

| | Private. | | | Pauper. | | | Total Lunatics. | Found Lunatic by Inquisition. | | | Criminal. | | | Chargeable to Counties or Boroughs. | | |
|---|---|---|---|---|---|---|---|---|---|---|---|---|---|---|---|---|
| | M. | F. | Total. | M. | F. | Total. | | M. | F. | Total. | M. | F. | Total. | M. | F. | Total. |
| Asylums . . . | 120 | 112 | 232 | 3,150 | 3,758 | 6,908 | 7,140 | 4 | 1 | 5 | 117 | 31 | 148 | 359 | 445 | 804 |
| Hospitals . . | 412 | 453 | 865 | 175 | 168 | 343 | 1,208 | 14 | 3 | 17 | 5 | – | 5 | 14 | 12 | 26 |
| Metropolitan Licensed Houses . . | 574 | 546 | 1,120 | 737 | 1,088 | 1,825 | 2,945 | 59 | 37 | 96 | 25 | 7 | 32 | 38 | 50 | 88 |
| Provincial Licensed Houses . . | 800 | 757 | 1,557 | 1,106 | 1,123 | 2,229 | 3,786 | 76 | 44 | 120 | 64 | 15 | 79 | 66 | 52 | 118 |
| Totals . . | 1,906 | 1,868 | 3,774 | 5,158 | 5,137 | 11,305 | 15,079 | 153 | 85 | 238 | 211 | 53 | 264 | 477 | 559 | 1,036 |

Since the last Report, licences have been granted to the following gentlemen:—Mr. Elliot, Munster House, for 35 patients; Dr. W. Conolly, Hayes Park, 10 male and 10 female patients; Dr. J. G. Davey, Vine Cottage, 5 female patients. Licences have been renewed to Beaufort House, Fulham, formerly licensed to Mr. Wing; a house on Turnham Green Terrace; and a licence has been granted conjointly to Messrs. Gregory and Bascome, Wyke House, in lieu of Dr. Costello. Dr. Tuke has become co-proprietor with Mrs. Tuke, of Manor House, Chiswick.

In the provinces—

"'Darnall Hall,' near Sheffield, in the county of York, formerly licensed to Mr. John Kitching.

"The 'Hull and East Riding Refuge,' in the county of York, formerly licensed to Mr. Casson, and now converted into the 'Hull Borough Asylum.'

"'Burgh Hall,' near Chorley, in the county of Lancaster, formerly licensed to Mr. J. Seed; and

"'Portland House,' at Halstock, in the county of Dorset, formerly licensed to Mr. Mercer."

The following houses have been licensed for the first time, viz. :—

"'Claxton Grange Retreat,' near York, has been licensed to Mr. John Jackson, for private and pauper patients; and

" 'Field House,' at Anlaby, in the East Riding of the county of York, has been licensed to Mr. Edward Casson, for the reception of private and pauper patients. [This house was licensed shortly before the date of our last Annual Report.']"

During the past year three additional public Asylums have been opened, viz. :—

" 'The Hull Borough Asylum,' lately called the 'Hull and East Riding Refuge,' and then licensed to Mr. Casson.

" The 'Birmingham Borough Asylum;' and

" The 'Manchester Royal Lunatic Hospital.' "

It gratifies us to hear the Commissioners observe, with regard to their official entries into the books kept at the asylums, that—

" Their general tenor has been favourable, and they have now the satisfaction of reporting that the various Institutions for the insane throughout the country are in an improved state, and that the conduct of the superintendents, officers, and attendants, in reference to the treatment of patients and the management of lunatic establishments, is for the most part humane and judicious."

It appears from the Report, that the Commissioners have at different times, during the past year, authorized the temporary removal of eighty-eight patients from various hospitals and licensed houses, within the metropolitan district, to the sea-side, or on visits to their friends, for limited periods, for the benefit of their health. They continue to believe that the authority conferred by this section has been found most useful, not only as contributing to the comfort and health of the patients, but as affording a means of testing the progress of their recovery, especially in those cases where the commissioners did not feel themselves justified in ordering their discharge, until they had undergone some previous probation under the immediate eye of their relatives or friends.

On the subject of suicides the Report states :—

" We have the satisfaction of stating that the deaths by suicide during the last year have amounted to only *eight in number*. Considering that the total number of lunatics in asylums in the country is not less than 15,000, and that the ancient system of mechanical restraint has in many institutions been altogether abandoned, and in most others exceedingly diminished, we cannot but consider that the number of deaths by suicide is smaller than might have been anticipated, and that the fact is highly creditable to the superintendents, medical officers, and attendants of the various establishments for the insane, to whose vigilance and care this result must be mainly attributed.

" Of the suicides referred to, six, viz. three males and three females, were by strangulation; one male by cutting his throat, and a female by drowning. In the last-mentioned case, which occurred in the metropolitan district, the lady referred to escaped from her attendant while walking with her in the country, secreted, and afterwards drowned herself.

"In every case of suicide we have required full particulars as to the place, time of day or night, and other circumstances; by what instrument or means the act was committed, and by whom, and after what period it was discovered.

"These inquiries are made with a view to ascertain whether or not any blame is imputable to attendants or others, and whether all proper arrangements had been previously made and precautions taken to prevent such an act, having regard to the particular case, more especially where the patient was known to have exhibited suicidal propensities. In two instances, which appeared to call for more than ordinary investigation, the local visitors, at the suggestion of this Board, instituted minute inquiries on the spot."

In the course of the Report allusion is made to the celebrated Nottidge and Ripley case. In illustration of the danger which would have accrued, had the Lord Chief Baron's dictum been acted upon, it is observed, as the result of a careful analysis of the patients in the Lancaster Asylum (made with reference to this subject, in the month of September last), that according to the dictum in question, upwards of 300 insane patients, totally unfit to take care of themselves, must have been turned loose upon society !

The Commissioners observe :—

"Whilst making our visitations in the course of the past year we had reason to believe that, in some instances, private patients in licensed houses had not the benefit of that suitable accommodation and those comforts to which they were entitled from their circumstances and situation in life. In some cases it appeared on inquiry that the relations were unable to afford a remuneration adequate to the expenditure necessary for proper accommodation and treatment, and in others that they neglected to appropriate a sufficient part of the patient's income in promoting his cure or adding to his general comforts.

"A few cases also came under our observation in which it was evident that the sums paid were amply sufficient to provide everything necessary for the comfort and restoration of the patients, but the benefit of which the patients in fact did not enjoy. A marked instance of the disproportion between the amount paid and the accommodation provided for a gentleman of property having been brought before the board, we issued the following circular to the proprietors of private asylums in the metropolitan district :—

"*Office of Commissioners in Lunacy,*
19, *New-street, Spring Gardens,* 12*th February,* 1850.

"'SIR,—The commissioners in lunacy having reason to suppose that in some cases a smaller allowance is made for the maintenance of patients in lunatic asylums than their annual income would justify; and also, that in other cases the amount of accommodation and comfort supplied to the patients is less than was stipulated for by the relatives, and less than, having regard to the sums charged, the patients may be considered to be reasonably entitled to, are desirous, and request that you will, without delay, make out a tabular list (according to the annexed form), specifying the names of all the private patients in your house (as far as may be alphabetically), separating the males from the females, and specifying also against their several names their stations or professions in life, together with the total annual rate of payment agreed to be charged for them respectively for their maintenance and treatment, and also for extras (if any); and the commissioners further request that you will have this list ready, and accessible, to them, whenever they may visit your licensed house.

" 'I am, &c.,

(Signed) " 'R. W. S. Lutwidge,

" 'To the Superintendent of ———.' Secretary.'

" We think it due to some proprietors of licensed houses to state that we have ascertained that in various instances superior comforts and accommodation have been afforded to patients, more with reference to their former habits and station in life than to the mere amount of money received for their maintenance."

The commissioners direct the attention of government to the necessity of adopting some different arrangements with reference to the disposal of criminal lunatics. They observe that the construction of lunatic asylums is so essentially different from that of prisons, that an effectual security against the escape of criminals cannot be provided for without restricting the liberty of other patients, with whom they are necessarily associated, and materially interfering with that treatment and general arrangement which ought to be adopted for their benefit.

Criminal patients have therefore escaped, and must continue to escape from asylums and houses licensed for the reception of the insane. As an instance of this they mention the fact, which was brought specially under the notice of secretary Sir George Grey, that a most active and cunning criminal patient escaped for the fifth time, from Hoxton House, in February last. The objection of the commissioners applies especially to such lunatics as have been charged with the more heinous offences; and it has been frequently brought under their notice that the friends and relatives of patients, and also the patients themselves when conscious of their being associated with criminal lunatics, have considered such association as a great and unnecessary aggravation of their calamity.

A passing allusion is made to the defective state of the law with reference to the property of lunatics; and a hope is expressed, that the matter will receive the Lord Chancellor's early attention. We think this subject one entitled to every consideration. Upon the whole, we consider this report extremely satisfactory to those connected with the care and cure of the insane. We hope it will have the effect of encouraging them in the exercise of their arduous, anxious, responsible, and often dangerous duties. Too much praise cannot be bestowed upon those who devote their time, abilities, and knowledge to the cure and amelioration of the condition of the insane. Every allowance should be readily made for the little defects that occasionally flit across the sun's disk; and the most friendly feeling should be encouraged between the proprietors of asylums and those officially engaged in their visitation and inspection. The first point is to attach men of respectability, attainments, and knowledge to the *personal management of the insane.* This would be the most effectual and the only mode of elevating the character of private lunatic asylums, and of removing a prejudice which, to some extent, continues to exist in the public mind against these institutions. Once this is effected, and the feeling prevails that entire confidence is to be placed in the medical proprietors of establishments for the insane, there will be a greater disposition to send friends and relatives, at an early and more curable stage of insanity, to private asylums; and it would effectually put an end to that system, so destructive to the integrity of the human mind, called "home" and "cottage" treatment.

---

